# Associations of *IDUA* and *PTCH1* with Bone Mineral Density, Bone Turnover Markers, and Fractures in Chinese Elderly Patients with Osteoporosis

**DOI:** 10.1155/2019/9503762

**Published:** 2019-06-02

**Authors:** Qifei Wang, Chong Tang, Junxiu Jia, Guangwu Zhang, Zheng Liu

**Affiliations:** Department of Orthopedics, Peking University Shougang Hospital, Shijingshan District, Beijing, China

## Abstract

**Introduction:**

Osteoporosis (OP) is a common polygenic disorder in the aging population, and several single nucleotide polymorphisms (SNPs) in the alpha-L-iduronidase (*IDUA*) gene and patched homolog 1 (*PTCH1*) gene regulate bone metabolism and affect bone mass. The study aimed at investigating the relationships of rs3755955 and rs6831280 in the *IDUA* gene and rs28377268 in the *PTCH1* gene with bone mineral density (BMD), bone turnover markers (BTMs), and fractures in the elderly Chinese subjects with OP.

**Materials and Methods:**

A cohort of 328 unrelated senile osteoporosis (SOP) patients with or without osteoporotic fractures was recruited. rs3755955, rs6831280, and rs28377268 polymorphisms were identified using SNaPshot technology. BTM levels were determined by electrochemiluminescence (ECL). Bone mineral densities (BMDs) at the lumbar spine (LS) and proximal femur sites were measured by dual-energy X-ray absorptiometry (DEXA) in all subjects. The Hardy-Weinberg equilibrium (HWE) test was performed. HWE *P* values and comparisons of genotype frequencies were estimated using the chi-square test. Analysis of covariance (ANCOVA) adjusted for confounding factors was performed to investigate associations of SNPs with BMDs and BTMs in subgroups.

**Results:**

The chi-square test indicated that genotype distributions in the control group conformed to HWE (*P* > 0.05). The distributions of allele and genotype frequencies of rs6831280 between fracture and osteoporotic participants were significantly different (P-allele = 0.002 and P-genotype = 0.012, respectively). Concerning rs6831280, ANCOVA found BMDs at LS 2-4 (L2-4) and total hip (TH) among the study subjects suffering from SOP with GA genotype were lower than in those carrying GG or AA (P-L2-4 = 0.004 and P-TH = 0.027, respectively).

**Conclusions:**

*IDUA* rs6831280 is associated with BMDs at L2-4 and TH in the elderly Chinese population with SOP and may serve as a marker for the genetic susceptibility to osteoporotic fractures.

## 1. Introduction

OP is a complex and polygenic disorder characterized by decreased bone mass, microarchitectural deterioration of the bone tissue, and elevated bone fragility and susceptibility to fractures [1]. Previous studies on twins and families indicated that genetic variants played an essential role in regulating bone metabolism and influencing bone mass [2]. It is estimated that the genetic factor accounts for approximately 60-80% of individual variance in BMD [3], which is a known predictor of the risk of osteoporotic fractures [4, 5]. Osteoporotic fractures, the most serious complication of OP in the elderly, are associated with poor prognosis. For instance, mortality rate in senile patients who suffer from hip fractures may reach as high as 20% in the first year after fractures [6, 7]. Hence, osteoporotic fractures diminish the quality of life and produce a heavy economic burden on individuals and healthcare systems [8].

Genome-wide association studies (GWAS) of common sequence variants in large-scale populations have in recent years discovered considerable common genetic variations that are related with BMD variants [9–11]. Accordingly, associations of SNPs and BMD or BTMs have become a research hotspot for the years ahead. For instance, SNPs such as rs6831280 and rs3755955 in the *IDUA* gene were reported to affect OP risk by protein phosphorylation that regulates a wide variety of cellular processes including activities of osteocytes [9]. For the IDUA protein, a predicted phosphorylation site T366 and two predicted phosphorylation sites T98 and S102 are indirectly affected by *IDUA* rs6831280 and rs3755955, respectively [9]. In addition, the *PTCH1* gene encodes receptors for the Indian Hedgehog (IHH), Sonic Hedgehog (SHH), and Desert Hedgehog (DHH). The Hedgehog-Patched 1 signaling is involved in homeostatic osteoblast activity and regulates bone remodeling [12]. Based on functional predictions of the HaploReg [13] and Roadmap Epigenomics Program [14] resources, rs28377268 was located in a region that overlaps with promoter histone marks in a variety of tissues and enhancer histone marks in multiple organs, which could be a protein-binding site and a DNase hypersensitivity site in osteoblasts. Furthermore, correlation of rs28377268 in the *PTCH1* gene with reduced spine BMD and osteoporotic fractures was previously reported [12].

Although the above SNPs were associated with BMD in previous studies, the conclusion may not apply to other ethnicities and populations due to genetic variations and diverse environmental factors. Besides, most previous studies involved women, especially postmenopausal women, instead of the elderly whose life quality would be seriously impaired when they suffer from osteoporotic fractures. What is more, most previous studies of association between SNPs and BMD did not focus on fractures, the main endpoint of OP. Our study aimed to investigate the relationships of rs3755955 and rs6831280 in the *IDUA* gene and rs28377268 in the *PTCH1* gene with BMDs, BTMs, and fractures in an elderly Chinese population.

## 2. Materials and Methods

### 2.1. Subjects

This was a cross-sectional study of a total of 328 SOP patients enrolled at the Beijing University Shougang Hospital. Among them, 172 elderly OP subjects suffering from fractures under low trauma were regarded as the fracture group which was composed of 103 females and 69 males, aged 65-96 years, with a mean of 76.9 ± 7.2 years. The remaining 156 participants with only OP were considered as the osteoporotic group which consisted of 98 females and 58 males, aged 65-90 years, with a mean of 72.4 ± 7.3 years. These two groups were frequency matched in age (±4.5 years), gender, and body mass index (BMI) (±2.111 kg/m^2^). In light of the World Health Organization (WHO) criteria, participants with *T*-score at the femoral neck (FN) or LS≤−2.5 were viewed as OP. SOP, formerly known as OP type II, is defined as primary OP in females aged >65 years or males aged >70 years. A detailed description of characteristics of the study participants is presented in Table 1. Clinical examinations and routine biochemical tests were performed to rule out patients with systemic or metabolic bone diseases, such as cardiovascular, hepatic, or renal disorders and secondary OP. None of the study participants had previously taken any drug known to interfere with bone metabolism. In addition, the study design accorded with the principles of the Declaration of Helsinki. The study was approved by Research Ethical Committee of Beijing University Shougang Hospital, and written informed consent was obtained from all subjects before their enrollments.

### 2.2. BTM Measurement

Blood samples of all participants were extracted from cubital veins in the morning. BTMs including *β*-CTX and PINP were tested using ECL assay kits from Roche Laboratory (Mannheim, Germany) according to the manufacturer's instructions. The entire measurement was accomplished by clinical laboratory physicians.

### 2.3. BMD Measurement

BMDs at the femoral trochanter (FT), FN, Ward's triangle (WT), total hip (TH), and LS2-4 were identified in all subjects using DEXA (QDR-4500; HOLOGIC Inc., Bedford, MA, USA). In addition, BMI (kg/m^2^) was calculated using body height and weight measured. The instrument was calibrated daily in accordance with the manufacturer's instructions. BMD values were described as grams per cm^2^. The criterion used for diagnosis of OP was the *T*-score at the FN or LS≤−2.5.

### 2.4. Genotyping

Genomic DNA was extracted from peripheral blood leukocytes obtained from subjects using the QIAamp DNA Blood Mini Kit (QIAGEN GmbH, Hilden, Germany). DNA was stored at -80°C until genotyped. SNPs were determined using SNaPshot technology, the best genotyping method except for the gold standard which is direct sequencing. The primers of rs3755955, rs6831280, and rs28377268 SNPs analyzed in the study were synthesized by the Shanghai Biological Engineering Technology Co. Ltd. The detailed information of primers including the forward primer and reverse primer is presented in Table 2. A random sample (5% of the total genotyped samples) was also genotyped in a separate control plate to ensure genotyping quality, and the coincidence rate was 100% between these results. Genotyping was completed by the Beijing Microread Gene Technology Co. Ltd.

### 2.5. Statistical Analysis

Continuous variables are expressed as the mean ± standard deviation (SD), and categorical variables are presented as frequencies and percentages. A software package in R called “HardyWeinberg” (https://cran.rproject.org/web/packages/HardyWeinberg/HardyWeinerg.pdf) was applied for performing the HWE test. A *P* value > 0.05 indicated HWE. The Kolmogorov-Smirnov and Levene tests were applied to investigate normal distribution and homogeneity of variance, respectively. Categorical variables between groups were examined by the *χ*^2^ test. Between-group differences with respect to normally distributed continuous variables were assessed with ANCOVA adjusted for confounding factors. Bonferroni correction was performed to control for multiple testing. Those with respect to nonnormally distributed variables were assessed with the nonparametric Kruskal-Wallis test. *P* < 0.05 was considered statistically significant. Statistical analysis was completed using SPSS software version 23.0.

Power Analysis and Sample Size (PASS) 14.0 was performed to estimate the sample size in our study design. The power for the primary endpoint BTM level and BMD value was calculated based on ANOVA with a significance level of 0.05. Main effect sizes such as admissible error (*δ*) and total population standard deviation (*σ*) were obtained on the basis of related literature previously published. With the confidence level *α* = 0.05 and the power 1-*β* = 0.80, we estimated the minimum sample size (143 patients each group) by using PASS.

## 3. Results

### 3.1. Basic Characteristics of the Study Sample

The detailed description of the study sample enrolled in the study is presented in Table 1. A cohort of 328 unrelated subjects, including 172 patients with osteoporotic fractures and 156 patients with OP, was recruited in the current research. Patients with osteoporotic fractures have significantly lower BMD values at the FT, WT, TH, and LS 2-4 compared with those with only OP (*P* < 0.05 for all with adjustment for age and BMI by ANCOVA). However, no basic features including age, gender, BMI, BTMs, and BMD value at FN indicated a significant difference between the two groups (*P* > 0.05 for all with adjustment for age and BMI by ANCOVA excluding age and gender).

### 3.2. The Distributions of Allele and Genotype Frequencies

The distributions of allele and genotype frequencies in subgroups are summarized in Table 3. The genotype distributions in the control group agreed closely with HWE. The allele and genotype frequency distributions of rs6831280 between the fracture and osteoporotic groups were significantly different (df-allele = 1, df-genotype = 2, *P* < 0.05 using the *χ*^2^ test). The fracture risk of AA was higher than that of GG or GA (df = 1, *P* < 0.05 using the chi-square test). However, there was no difference in the fracture risks of GG and GA (df = 1, *P* > 0.05/3 = 0.0167 using Bonferroni correction). No statistically significant difference was detected in the distributions of allelic and genotypic frequencies of rs3755955 and rs28377268 between the two groups (df-allele = 1, df-genotype = 2, *P* > 0.05 using the *χ*^2^ test).

### 3.3. Associations of SNPs with BTMs and BMD in Subgroups

The relationships of SNPs with BTMs and BMD among the elderly with osteoporotic fractures are shown in Table 4. No significant associations between the genotyped SNPs and basic characteristics were observed after adjustment for age and BMI by ANCOVA (*P* > 0.05).

The associations of rs3755955, rs6831280, and rs28377268 with BTMs and BMD among the study participants with OP are presented in Table 5. With regard to SNP rs6831280, the BMDs at L2-4 and TH in the OP group with the GA genotype were lower than in those carrying GG or AA after adjustment for age and BMI by ANCOVA (*P* < 0.05). However, in terms of rs3755955 and rs28377268, no significant differences in BTM levels and BMD at any of the skeletal locations were found among osteoporotic patients after adjustment for age and BMI by ANCOVA (*P* > 0.05).

## 4. Discussion

The study aimed to investigate the associations of rs3755955 and rs6831280 in the *IDUA* gene and rs28377268 in the *PTCH1* gene with BMD, BTMs, and fractures in a cohort of 238 unrelated SOP with or without osteoporotic fractures. Importantly, the BMDs at L2-4 and TH in patients suffering from OP with the GA genotype of rs6831280 were lower than in those carrying the GG or AA genotype. In addition, we detected that the distributions of allele and genotype frequencies of rs6831280 between the fracture and osteoporotic groups were significantly different.

Primary OP, a major public health issue worldwide, consists of postmenopausal OP (PMOP; type I) and SOP (type II) [15]. Dissimilar to PMOP typically occurring 5–10 years after menopause mainly due to decline in estrogen levels, SOP in which the bone mass is affected by ageing instead of sex hormone deficiency generally refers to OP in females aged >65 years or males aged >70 years [16, 17]. Individuals with SOP are more inclined to suffer from osteoporotic fractures under low-energy injury which raise the morbidity and mortality in the elderly population. The etiology of SOP is mainly influenced by environmental and genetic factors [18]. Numerous GWAS and candidate gene studies have demonstrated that genetic variances in the vitamin D receptor (*VDR*) gene [19, 20], lipoprotein receptor-related protein 5 (*LRP5*) gene [21–24], and Insulin-like Growth Factor-1 (*IGF-1*) gene [25–27] are involved in the regulation of bone remodeling and affect bone mass. In addition, the *IDUA* gene and the *PTCH1* gene have been a research focus on the relation of SNPs with BMD and OP in recent years. While the relationships of rs3755955 and rs6831280 in the *IDUA* gene and rs28377268 in the *PTCH1* gene with decreased spine BMD and osteoporotic fractures were previously detected [9, 12], the conclusion may not apply to other ethnicities and populations mainly ascribed to the heterogeneity of study populations. Accordingly, further studies are required to investigate their associations with BMD, BTMs, and fractures in other nationalities and regions, especially in the population with SOP.

The genotype distributions of rs3755955, rs6831280, and rs28377268 in the control group conformed to HWE (*P* > 0.05 for all). Interestingly, there were considerable differences in the distributions of rs6831280 alleles and genotypes between two groups (P-allele = 0.002; P-genotype = 0.012). The AA genotype frequency in the fracture group was significantly higher than that in the osteoporotic group, which was a good predictor for fracture risk. Different from allele and genotype frequencies of rs3755955 in the HapMap Project investigating 845 subjects (16.9% A, 83.1% G, and 2.85% AA, 28.04% AG, and 69.11% GG), the allelic and genotypic frequencies in the study were 22.6% A, 77.4% G, and 7% AA, 31.1% AG, and 61.9% GG.(P-allele = 0.001, P-genotype = 0.002). The difference can be explained by diversities of ethnicities and populations. The rs6831280 genotype frequencies in all participants were 62.4% GG homozygotes, 32.1% GA heterozygotes, and 5.5% AA homozygotes, with 78.5% G and 21.5% A allele frequencies, respectively. The G and A allele frequencies of rs6831280 described in the HapMap Project investigating 1090 participants are 78.2% and 21.8%, respectively. No significant difference in allele and genotype frequencies of rs6831280 was found between our study and the HapMap Project (P-allele = 0.374, P-genotype = 0.739). With regard to rs28377268, AA, AC, and CC genotype frequencies in the whole study sample were 7.7%, 47.3% and 45.0%, respectively, with allele frequencies 31.4% A and 68.6% C. The A and C alleles expressed in the HapMap Project were 24.0% and 76%, respectively, in a cohort of 1203 subjects. No significant difference in allele and genotype frequencies of rs28377268 was found between our study and the HapMap Project (P-allele = 0.114, P-genotype = 0.218). Consistency in the allele and genotype frequencies of the present study and the HapMap Project indicated no remarked difference in genetic variations of the two SNPs. In addition, the detailed description of allele and genotype distributions of rs3755955, rs6831280, and rs28377268 was not presented in previous studies [9, 12].

One point to illustrate is that SOP patients were females aged >65 years or males aged >70 years and they had similar etiology, namely, ageing rather than sex hormone deficiency. In addition, no significant difference in gender between subgroups was found (*P* = 0.586); hence, we integrated females and males into one subgroup. In the study, participants with osteoporotic fractures had significantly lower BMDs at the studied sites except for FN compared to those with OP (*P* < 0.05 for all). Genetic variations, age, BMI, lifestyle, and physical activities may explain the distinct differences in BMD values between these two groups. But no basic features including age, gender, and BMI showed a significant difference among subgroups (*P* > 0.05 for all). Furthermore, no significant difference in BTM levels among subgroups was detected (*P* > 0.05 for all).

We did not observe significant relationships of the analyzed SNPs with BTMs and BMD in subjects with osteoporotic fractures. This did not exclude the possibility of the association with clinical straits in that the effect seemed to be too slight to be identified in our study sample with osteoporotic fractures. Niu et al. reported that *IDUA* rs3755955, a Type II (+) PhosSNP, was associated with BMD-FN, BMD-TH, and BMD-LS with *P* = 8.36^∗^10^−10^, 3.26^∗^10^−6^, and 9.50^∗^10^−3^, respectively [9]. Rs28377268 was reported to associate with reduced spine BMD and osteoporotic fractures in a genome-wide association study of BMD among 20,100 Icelanders and 10,091 participants of European and East-Asian descent [12]. However, we did not find relationships of rs3755955 and rs28377268 with BTM levels and BMD values in our study. The finding was not completely consistent with the report of Niu et al. [9], which could be attributed to genetic heterogeneities in different study populations, population admixture, and gene-environment or gene-gene interactions. In addition, rs6831280 was demonstrated to be associated with the BMD phenotype among individuals with SOP in our study. The BMDs at L2-4 and TH in SOP patients with the GA genotype were lower than in those carrying GG or AA (P-L2-4 = 0.004, P-TH = 0.027, respectively). Although rs6831280 was associated with BMD in our study and published literature, previous findings validated *IDUA* rs6831280 to be significantly associated with BMD-FN. Different BMD measurements, lifestyle, and time of exposure to sunshine are responsible for association with different sites observed in our study and previous data.

There are several potential limitations in our study. To begin with, the sample size in the study was modest. Consequently, it may limit the statistical power to detect genotype-phenotype association and genetic influence on the development of SOP. Secondly, the SNPs were not investigated from GWAS, but rather selected from the previous studies. For the Hedgehog signaling pathway, besides the *PTCH1* gene, there are other genes in this important pathway for bone development that could harbor molecular variants, such as the *SHH*, *PTCH2*, and *SMO* genes [28, 29], and genetic polymorphisms located on these candidate genes could be investigated in the future. It must be pointed out that the aged without OP were not recruited because there were so few individuals with normal bone mass or osteopenia especially aged above 75 years who can match the fracture or osteoporotic group in age and BMI. Furthermore, we did not collect information on dietary calcium and vitamin D intake and lifestyle-related variables such as alcohol intake, smoking, and physical activity levels in the analysis. Additionally, our study only assessed single-SNP effects. However, single-SNP association studies could be more informative than haplotype-based association studies [23, 30]. Accordingly, the association of haplotypes formed by SNPs on a clinical phenotype could be assessed in a future study.

## 5. Conclusions

A statistically significant association between rs6831280 and BMDs at L2-4 and TH was observed in the study. The distributions of rs6831280 allele and genotype frequencies were found to be significantly different between the osteoporotic and fracture groups.

## Figures and Tables

**Table 1:** Characteristics of participants disaggregated by study group.

Variable	Fracture group	Osteoporotic group	df	*P*
	Mean ± SD	Mean ± SD		
Age (years)	76.9 ± 7.2	72.4 ± 7.3		0.242
Female/male	103/69	98/58	1	0.586
BMI (kg/m^2^)	19.712 ± 2.327	21.823 ± 2.472		0.082
*β*-CTX (ng/ml)	0.455 ± 0.315	0.458 ± 0.254		0.947
PINP (ng/ml)	53.421 ± 30.379	49.941 ± 31.307		0.487
BMD-L2-4 (g/cm^2^)	0.770 ± 0.150	0.988 ± 0.277		<0.001
BMD-FN (g/cm^2^)	0.595 ± 0.131	0.614 ± 0.198		0.098
BMD-WT (g/cm^2^)	0.407 ± 0.243	0.521 ± 0.178		0.009
BMD-FT (g/cm^2^)	0.525 ± 0.128	0.651 ± 0.155		<0.001
BMD-TH (g/cm^2^)	0.719 ± 0.209	0.836 ± 0.167		0.003

BMI: body mass index; *β*-CTX: procollagen type I carboxy terminal peptide beta special sequence; PINP: procollagen I N-terminal propeptide; BMD: bone mineral density; L2-4: L2-4 vertebra; FN: femoral neck; WT: Ward's triangle; FT: femoral trochanter; TH: total hip.

**Table 2:** PCR primers for SNPs genotyped.

Polymorphic loci	Forward primer (5′-3′)	Reverse primer (5′-3′)	Extent (bp)
rs3755955	CGCAGCATCAGAACCTGCTACT	CGGG(T/G)G(T/C/G)TGTTGACCTGGAAG	215
rs6831280	TCTGAAACTGTCCTGTTGACTCAG	ATCAATGTTGAGAGCAATTGTCAG	360
rs28377268	TGTGCACGCAGGGAAATAC	TCTCTTTACAGCTGCAGCC	244

**Table 3:** Distributions of allele and genotype frequencies in the study sample.

SNP	Group	Allele		df	P-Allele	Genotype			df	P-Genotype	P-HWE
		G	A			GG	GA	AA			
rs3755955	Fracture	264 (76.7%)	80 (23.3%)	1	0.709	105 (61.0%)	54 (31.4%)	13 (7.6%)	2	0.902	
	Control	244 (78.2%)	68 (21.8%)			98 (62.8%)	48 (30.8%)	10 (6.4%)			0.224
	Total	508 (77.4%)	148 (22.6%)			203 (61.9%)	102 (31.1%)	23 (7.0%)			

		G	A			GG	GA	AA			
rs6831280	Fracture	254 (73.8%)	90 (26.2%)	1	0.002	95 (55.2%)	64 (37.2%)	13 (7.6%)	2	0.012	
	Control	264 (83.5%)	52 (16.5%)			111 (70.2%)	42 (26.6%)	5 (3.2%)			0.676
	Total	518 (78.5%)	131 (21.5%)			206 (62.4%)	106 (32.1%)	18 (5.5%)			

		A	C			AA	AC	CC			
rs28377268	Fracture	73 (21.2%)	271 (78.8%)	1	0.924	9 (5.2%)	55 (32.0%)	108 (62.8%)	2	0.992	
	Control	65 (20.8%)	247 (79.2%)			8 (5.1%)	49 (31.4%)	99 (63.5%)			0.551
	Total	138 (21.0%)	518 (79.0%)			17 (5.2%)	104 (31.7%)	207 (63.1%)			

**Table 4:** Associations of SNPs genotyped with BTMs and BMDs in participants with osteoporotic fractures.

Variable	rs3755955			*P*	rs6831280			*P*	rs28377268			*P*
	CC	CT	TT		GG	GA	AA		AA	AC	CC	
*β*-CTX (ng/ml)	0.484 ± 0.383	0.447 ± 0.302	0.325 ± 0.163	0.418	0.476 ± 0.332	0.449 ± 0.315	0.335 ± 0.158	0.387	0.355 ± 0.166	0.414 ± 0.257	0.483 ± 0.348	0.438
PINP (ng/ml)	57.117 ± 36.236	50.882 ± 32.354	39.398 ± 14.827	0.312	56.227 ± 30.237	52.18 ± 33.683	38.348 ± 13.879	0.215	40.150 ± 13.001	52.009 ± 32.242	54.972 ± 30.437	0.551
BMD-L2-4 (g/cm^2^)	0.800 ± 0.180	0.759 ± 0.111	0.800 ± 0.179	0.695	0.772 ± 0.176	0.739 ± 0.099	0.927 ± 0.165	0.136	0.661 ± 0.080	0.800 ± 0.081	0.773 ± 0.174	0.499
BMD-FN (g/cm^2^)	0.606 ± 0.131	0.610 ± 0.142	0.718 ± 0.142	0.722	0.572 ± 0.118	0.602 ± 0.134	0.697 ± 0.187	0.307	0.481 ± 0.034	0.617 ± 0.112	0.596 ± 0.143	0.418
BMD-WT (g/cm^2^)	0.398 ± 0.144	0.452 ± 0.323	0.418 ± 0.224	0.767	0.360 ± 0.120	0.451 ± 0.334	0.442 ± 0.210	0.543	0.256 ± 0.072	0.385 ± 0.107	0.431 ± 0.295	0.601
BMD-FT (g/cm^2^)	0.536 ± 0.127	0.530 ± 0.136	0.628 ± 0.318	0.767	0.513 ± 0.122	0.529 ± 0.121	0.574 ± 0.238	0.747	0.444 ± 0.115	0.540 ± 0.115	0.528 ± 0.139	0.636
BMD-TH (g/cm^2^)	0.702 ± 0.141	0.747 ± 0.262	0.901 ± 0.314	0.545	0.677 ± 0.128	0.753 ± 0.264	0.776 ± 0.282	0.513	0.602 ± 0.079	0.733 ± 0.154	0.722 ± 0.241	0.728

*β*-CTX: procollagen type I carboxy terminal peptide beta special sequence; PINP: procollagen I N-terminal propeptide; BMD: bone mineral density; L2-4: L2-4 vertebra; FN: femoral neck; WT: Ward's triangle; FT: femoral trochanter; TH: total hip. All *P* values were adjusted for age and BMI by ANCOVA.

**Table 5:** Associations of SNPs genotyped with BTMs and BMDs in participants with OP.

Variable	Rs3755955			*P*	Rs6831280			*P*	Rs28377268			*P*
	CC	CT	TT		GG	GA	AA		AA	AC	CC	
*β*-CTX (ng/ml)	0.449 ± 0.251	0.513 ± 0.247	0.484 ± 0.280	0.44	0.429 ± 0.254	0.529 ± 0.256	0.418 ± 0.099	0.177	0.505 ± 0.397	0.461 ± 0.263	0.455 ± 0.243	0.914
PINP (ng/ml)	49.346 ± 30.700	44.854 ± 28.132	67.111 ± 100.792	0.285	46.276 ± 30.348	58.654 ± 30.573	47.100+ 25.738	0.392	34.300 + 20.422	57.074 + 38.869	47.189 + 30.893	0.44
BMD-L2-4 (g/cm^2^)	1.034 ± 0.270	0.987 ± 0.273	0.838 ± 0.532	0.448	1.037 ± 0.266	0.813 ± 0.231	0.931 ± 0.265	0.004^∗^	1.059 ± 0.187	0.968 ± 0.307	1.007 ± 0.263	0.837
BMD-FN (g/cm^2^)	1.252 ± 3.568	0.995 ± 1.223	0.685 ± 0.206	0.893	0.829 ± 0.184	0.971 ± 0.378	0.884 ± 0.050	0.549	0.806 ± 0.083	0.701 ± 0.124	0.882 ± 0.101	0.724
BMD-WT (g/cm^2^)	1.199 ± 4.592	0.567 ± 0.203	0.502 ± 0.179	0.758	0.532 ± 0.184	0.482 ± 0.165	0.683 ± 0.038	0.256	0.629 ± 0.243	0.488 ± 0.141	0.539 ± 0.191	0.413
BMD-FT (g/cm^2^)	0.668 ± 0.153	0.688 ± 0.195	0.631 ± 0.157	0.786	0.664 ± 0.149	0.603 ± 0.141	0.852 ± 0.173	0.053	0.654 ± 0.016	0.629 ± 0.122	0.669 ± 0.169	0.653
BMD-TH (g/cm^2^)	1.178 ± 2.233	0.829 ± 0.271	0.838 ± 0.247	0.711	0.863 ± 0.147	0.767 ± 0.179	1.032 ± 0.159	0.027^#^	0.834 ± 0.075	0.828 ± 0.136	0.848 ± 0.185	0.915

*β*-CTX: procollagen type I carboxy terminal peptide beta special sequence; PINP: procollagen I N-terminal propeptide; BMD: bone mineral density; L2-4: L2-4 vertebra; FN: femoral neck; WT: Ward's triangle; FT: femoral trochanter; TH: total hip. All *P* values were adjusted for age and BMI by ANCOVA. ^∗^GG vs. GA *P* < 0.001, GG vs. AA *P* = 0.026, and GA vs. AA *P* = 0.179 by post hoc Bonferroni test. ^#^GG vs. GA *P* = 0.329, GG vs. AA *P* = 0.039, and GA vs. AA *P* = 0.011 by post hoc Bonferroni test.

## Data Availability

The data used to support the findings of this study are available from the corresponding author upon request.
